# Antimicrobial Activity of Honey against Oral Microorganisms: Current Reality, Methodological Challenges and Solutions

**DOI:** 10.3390/microorganisms10122325

**Published:** 2022-11-24

**Authors:** Diego Romário-Silva, Severino Matias Alencar, Bruno Bueno-Silva, Janaína de Cássia Orlandi Sardi, Marcelo Franchin, Rafaela Durrer Parolina de Carvalho, Thayná Ellen de Sousa Alves Ferreira, Pedro Luiz Rosalen

**Affiliations:** 1 Department of Biosciences, Piracicaba Dental School, University of Campinas (UNICAMP), Piracicaba 13414-903, Brazil; 2 Research Program in Integrated Dental Sciences, University of Cuiabá, Cuiabá 78065-900, Brazil; 3 Department of Agri-Food Industry, Food and Nutrition, Luiz de Queiroz College of Agriculture (Escola Superior de Agricultura “Luiz de Queiroz”—ESALQ), University of São Paulo, Piracicaba 13418-900, Brazil; 4 Dental Research Division, Guarulhos University, Guarulhos 07023-070, Brazil; 5 School of Dentistry, Federal University of Alfenas (Unifal-MG), Alfenas 37130-001, Brazil; 6 Biological Sciences Graduate Program, Federal University of Alfenas (Unifal-MG), Alfenas 37130-001, Brazil

**Keywords:** honey, antimicrobial activity, antibiofilm activity, functional food, oral health, complementary therapy

## Abstract

Honey has been shown to have antimicrobial activity against different microorganisms, but its effects on oral biofilms are largely unknown. In this review, we analyzed the currently available literature on the antimicrobial activity of honey against oral biofilms in order to determine its potential as a functional food in the treatment and/or prevention of oral diseases. Here, we compare studies reporting on the antimicrobial activity of honey against systemic and oral bacteria, discuss methodological strategies, and point out current gaps in the literature. To date, there are no consistent studies supporting the use of honey as a therapy for oral diseases of bacterial origin, but current evidence in the field is promising. The lack of studies examining the antibiofilm activity of honey against oral microorganisms reveals a need for additional research to better define aspects such as chemical composition, the mechanism(s) of action, and antimicrobial action.

## 1. Introduction

Honey is a natural food produced by bees, mainly *Apis melífera*, composed mainly of sugars, water, proteins (enzymes), organic acids, vitamins, minerals, pigments, phenolic compounds, volatile compounds, solid particles derived from harvest, and an abundant amount and variety of phytochemicals [1,2]. Some honey constituents may play important biological roles in humans since the diversity of the biome(s) reached by the insects reflects in a direct fashion the wide bioactivity of the honey sample [3].

Honey has been used by mankind since ancient times, not only to meet nutritional needs but also as a medicine, which characterizes it as a functional food [4]. The most common reports of the medicinal use of honey were to treat small lesions, with an effect on healing and infectious processes [5]. The use of honey to treat wounds, bedsores, and other injuries was once a common practice that persisted in hospitals across Europe until the 1970s. The effectiveness of honey has been attributed to its biological properties, such as antimicrobial, anti-inflammatory, tissue repairing, deodorizing, and debridement of wounds. In recent years, modern medicine has rediscovered the therapeutic benefits of honey in the treatment of wounds, bedsores, and other conditions in hospitalized patients [6,7,8]. This has encouraged the exploration of the biological properties of honey against several medical conditions, such as biofilm-dependent oral diseases.

Several infectious diseases affecting humans are caused by biofilm-forming microorganisms, which include oral infections such as dental caries, periodontal diseases, and endodontic and fungal infections [9]. Bacteria that grow within a biofilm usually exhibit altered phenotypes, such as increased resistance to antimicrobial agents. Stable structural properties and proximity to bacterial cells in the biofilm favor horizontal transfer of resistance genes, which can ultimately increase antibiotic resistance rates [10]. This may render antimicrobial agents ineffective because, in addition to providing a facilitated environment for gene mutation, the biofilm scaffold forms a physical barrier against antimicrobial agents [10], which seems to be a crucial factor for the establishment and progression of microbial diseases [11].

While the antimicrobial and antibiofilm activities of different types of honey were previously determined against several systemic pathogens [5], little is known about its use as a functional food for the prevention and/or treatment of biofilm-dependent oral diseases. Evidence suggests that honey is not only an important source of energy, proteins, and minerals but also a potential functional food that may contribute to physical and mental well-being, preventing and mitigating risk factors for various diseases and improving/maintaining bodily functions [12].

Thus, the purpose of this review is to discuss the antimicrobial properties of honey as a functional food, with a special focus on biofilm-dependent oral diseases and the use of analytical prospecting methods.

## 2. Oral Microbiome

The oral microbiome is a complex and diverse community with more than 700 microbial species, which may be embedded in self-produced extracellular polysaccharides. The most common bacterial genera of the oral microbiome are *Veillonella, Actinomyces,* and *Streptococcus* [13,14]. These microorganisms can develop pathogenic biofilms on the surface of the teeth and cause oral diseases, such as caries and periodontal disease [15].

Dental biofilm can be classified into supragingival, located at or above the gingival margin, or subgingival, below the gingival margin, between the tooth and the gingival sulcular tissue [9]. Primary colonizers of supragingival dental plaque are predominantly facultative anaerobes (*Streptococcus* and *Actinomyces*), while subgingival areas are populated by strict anaerobes (*Bacteroidaceae* spp. and spirochetes) as a result of reduced oxygen availability [16].

Early biofilm formation and maturation take place through an interplay between bacteria–host, bacteria–bacteria, and bacteria–other microorganisms by means of various types of chemical bonds [17]. The wide range of chemical interactions between microbial cells, which are embedded in an extracellular polysaccharide matrix, is a complex and communicable network that provides a physical barrier and enables the expression and sharing of genes encoding for virulence factors. These conditions render biofilm pathogens approximately 1000-fold more resistant than their planktonic form. Thus, biofilm control represents the biggest challenge for the prevention and/or treatment of biofilm-dependent diseases [18].

**Figure 1:**
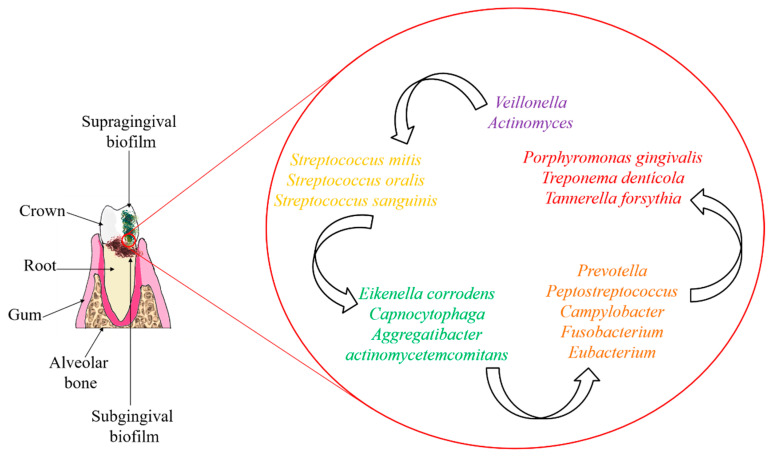
Schematics of the anatomical location of supragingival and subgingival biofilms, and the main bacterial species in each color-based complex.

Previously, mature subgingival biofilms were cataloged into different color-based complexes (purple, yellow, green, orange, and red), according to the frequency and quantity or abundance with which the microbial species are recovered from healthy patients and from those with periodontal disease [19,20] (Figure 1). The Sokransky diagram was based on the results of several clustering and sorting analyses of the microbiome with a considerable amount of biofilm samples (*n* = 185 individuals) by different clustering and sorting techniques [19]. Purple, yellow, and green complex microorganisms are associated with periodontal health, whereas orange complex bacteria are associated with health-disease dysbiosis, and red complex species are strongly associated with the onset of periodontal disease.

Currently, the most common periodontal treatment includes scaling and root planing (SRP). However, this procedure alone may be insufficient to eradicate oral pathogens from the subgingival environment. Thus, the use of antimicrobial therapy as an adjunct to SRP is frequently needed. Antimicrobials can be administered systemically or through a local delivery system. Several well-documented randomized controlled trials and systematic reviews have shown that the systemic use of amoxicillin plus metronidazole (in combination with SRP) produces ample clinical benefits when compared to SRP alone. However, the risk of selecting resistant microorganisms with mono drug therapy should be considered. Alternatively, natural products containing several bioactive molecules and systems acting synergistically could be considered an interesting antimicrobial treatment [21].

Natural resources are a promising source of antibiofilm agents. In recent years, plants and natural foods have been increasingly arousing the attention of the scientific community because of their health benefits. While most studies on the antibiofilm activity of natural products have focused on oral pathogens [22], the effectiveness of honey has been examined mostly against systemic microorganisms [5].

To be effective against dental biofilm, an antimicrobial or functional food should ideally act against most of these microorganisms, especially those in the form of biofilm, being a promising control of microbial colonization and oral biofilm formation.

## 3. What Explains the Antimicrobial Activity of Honey?

The broad-spectrum antibacterial activity of honey has previously been reported in the literature, but it still remains uncertain on some aspects, such as the elucidation of the bioactive components and mechanism(s) of action.

Thus far, the available evidence shows that there is no specific component responsible for the antimicrobial action of honey. Instead, its different compounds and characteristics seem to act synergistically, among which are low pH, osmotic effect, presence of hydrogen peroxide, phenolic compounds (mainly phenolic acids and flavonoids), methyl glyoxal, and bee peptides [23].

Overall, the antimicrobial mechanism of honey is mostly related to the production of peroxides by the enzymatic complex, particularly the enzyme glucose oxidase [24]. A brief description of each of these components and their relationship to the antimicrobial activity of honey can be found in Table 1. In addition, the induction of cytokine release in the host can also be an indirect antimicrobial mechanism of honey [23].

The biological properties of honey, including its antimicrobial activity, have been associated with the diverse chemical composition of this functional food. The phytochemical profile of honey is complex and can be influenced by different factors, such as geographic location, biodiversity of the local flora, as well as climatic conditions and seasonality [32]. While the phytoconstituents contained in the plant material collected by bees are also considered determinants of the antibacterial activity of honey [33], they remain poorly characterized [5]. Currently, several active components have already been identified in honey, but their inhibitory activity can no longer be attributed only to the common characteristics mentioned earlier [5], as other bioactive components are also likely to be involved.

Thus, studies examining the antimicrobial activity of honey should be mostly focused on determining its underlying mechanism(s) of action. According to antimicrobial mechanisms, honeys can be divided into two main groups, namely peroxide and non-peroxide honeys [34]. Overall, most honeys with proven antimicrobial activity are classified as peroxide honeys because their antimicrobial activity is linked to the production of hydrogen peroxide [34]. The most important non-peroxide honey representative is a New Zealand honey known as Manuka honey. It was designated as non-peroxide because even with the inactivation of these compounds, this honey exhibited significant antimicrobial activity. The hydrogen peroxide production in Manuka honey is relatively low. Nevertheless, Manuka honey has a potent antimicrobial activity, even after inactivating the peroxides with the enzyme catalase. Thus, the term “non-peroxide activity” (NPA) was proposed for that honey category. As a result, different samples of Manuka honey have been classified through their NPA unit [35,36,37]. Manuka honey properties are related to the presence of methylglyoxal [38].

Some types of honey are more potent than others, but all of them contain the same antibacterial substance (H_2_O_2_), except for Manuka honey. Recent evidence suggests that H_2_O_2_ present in honey is also produced via an alternative non-enzymatic pathway. In this sense, there is no correlation between the content of glucose oxidase and H_2_O_2_ levels. In addition, similarity has been demonstrated between minimum inhibitory concentration (MIC) values of diluted honeys untreated and treated with proteinase-K (protease). These findings suggest that the antimicrobial activity attributed to peroxides produced by the enzyme complex and defensin-1 is virtually negligible [28,39,40].

The answers to many questions about the antimicrobial mechanisms of honey remain unclear. Studies have reported that for H_2_O_2_ killing to occur, compound concentrations greater than 50 mM are required [41]. However, the H_2_O_2_ content in honey is 900 times less than that used in medical disinfectants [42]. A study demonstrated that when present in honey at a concentration of 1 mM, H_2_O_2_ was able to inhibit the growth of *Pseudomonas aeruginosa* and *Staphylococcus aureus* [39]. Furthermore, the authors suggested that the plant-derived polyphenolic compounds present in honey may be related to H_2_O_2_ production and, consequently, to the increase in the antibacterial potency of honey samples. A significant correlation between the concentration of total polyphenols and the antibacterial activity of honey samples was observed. The authors pointed out that the antimicrobial activity causally related to phenolic compounds is minimal and practically negligible since the concentration of polyphenols and flavonoids dissolved in honey is relatively low. In summary, the study suggested that the presence of phenolic compounds may be related to the antimicrobial activity of honey in two ways: H_2_O_2_ production and reduction of Fe (III) into Fe (II), triggering the Fenton reaction and producing more potent reactive oxygen species.

Other studies examining Polish honeys reported the importance of phenolic compounds for their antimicrobial activity [43,44,45]. The authors observed a small amount of these compounds in honey and suggested that a synergism between them and the H_2_O_2_-producing system is likely.

The conditions of matured honeys could resemble the macromolecular agglomeration in the living cell and affect the concentration, reactivity, and conformation of the macromolecules. Thus, a previous study evaluated the structure and distribution of honey components [46]. The authors found that a high concentration of macromolecules promoted the self-assembly of micron-sized superstructures. These structures were visible under a scanning electron microscope (SEM) as a biphasic system composed of dense globules dispersed in sugar. After diluting, these particles showed greater conformational stability. Moreover, at the threshold concentration, the system went through a phase transition with concomitant fragmentation of large micron-sized particles to nanoparticles in a hierarchical order. The authors concluded that the biphasic conformation of honey was needed to produce H_2_O_2_ and, consequently, that it determined the antibacterial activity of the sample. These properties disappeared beyond the phase transition point. This study suggests that the arrangement of active macromolecules with colloidal properties, organized in compact and stable multicomponent sets, is an important discovery about the overall structure of honey and is essential to understanding the complexity of the biological activities of such a functional food.

**Figure 2:**
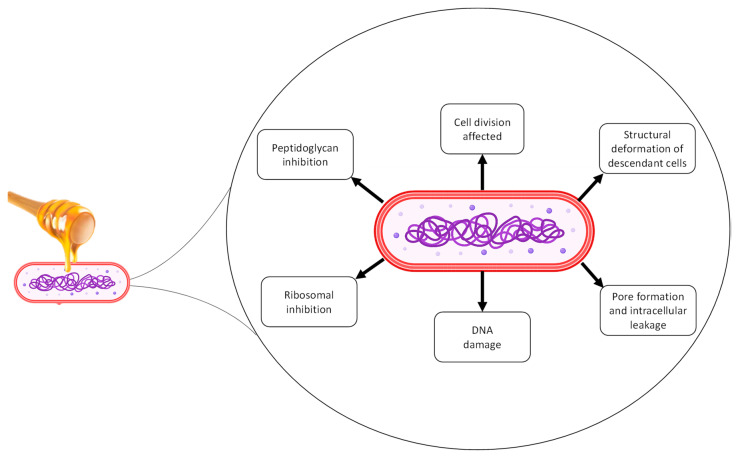
Mechanism of action of honey on bacteria.

Regarding the mechanism of action that honey exerts on bacterial cells, Manuka honey has been reported to have the ability to interfere with the septal ring in the process of cell division of *Staphylococcus aureus* [47]. The same effect was not observed in artificial honey used as a control [48]. In addition, it was also demonstrated that at sublethal doses, Manuka honey acted on *Bacillus subtilis* and *Staphylococcus aureus*, causing cells to shrink and the condensation of chromosomes [47]. Oxidative damage causing the inhibition of bacterial growth and DNA degradation in *Escherichia coli* and *Bacillus subtilis* has also been reported for honey with significant H_2_O_2_ content, although these effects have been modulated by other honey components [41]. Despite this, studies do not correlate chemical differences between phenolic compounds, floral origin, and collection time with the aforementioned mechanisms [49]. Figure 2 summarizes the main cellular damage from honey already reported on different types of bacteria.

Thus far, mounting evidence indicates that honey has a multifactorial antimicrobial activity consisting of the synergism between physical characteristics (reactivity and conformation of macromolecules) and chemical composition. It is unlikely that a specific isolated component of honey has an antimicrobial activity as good as that of its originating fresh honey. Hence, future research in the field should focus on understanding the mechanisms of action of whole honey, particularly its molecular targets. Detailed studies, including transcriptome analysis, can contribute to elucidating the global effects of honey on microbial cells [50].

## 4. Analysis of the Antimicrobial Activity of Honey

The antimicrobial activity of honey has been determined by two methods, namely agar diffusion and broth microdilution. However, the agar diffusion method has not been commonly recommended for most antibacterial substances since growth inhibition does not necessarily mean microbial death. Therefore, this method does not distinguish between bactericidal and bacteriostatic effects. In addition, it is not possible to measure the amount of substance that diffuses through the agar or the viscosity-related variability issues. These are some of the reasons why this method has not been indicated for determining the MIC [51].

Apart from the general considerations on the use of agar diffusion to determine the antimicrobial activity of any substance, there are particularities to consider. For instance, high viscosity and volume issues when placing honey samples into agar wells may render even more imprecise results. Another important point to consider is that the high molecular weight of active constituents present in honey may not diffuse properly through the agar (e.g., defensin-1 and especially glucose oxidase) [31]. Therefore, we recommend and discuss here only the use of broth microdilution to determine the antimicrobial activity of honey.

Dilution methods are the most suitable for determining MIC values. The MIC is defined as the lowest concentration of an antimicrobial that inhibits visible microbial growth. There are many approved guidelines for testing antimicrobial susceptibility using dilution methods. The most recognized standards are provided by the Clinical & Laboratory Standards Institute (CLSI) and the European Committee of Antimicrobial Susceptibility Testing (EUCAST). While the development of these standards does not guarantee the clinical relevance of the tests, it allows for the standardization and reproducibility of in vitro assays [52].

Broth microdilution involves preparing 1:2 dilutions of the antimicrobial agent in a liquid growth medium in a 96-well plate, i.e., 1000, 500, 250, and 125 µg/mL. This procedure is called serial dilution. The most problematic step in this test is to prepare a work solution with the honey sample. The first difference in relation to the preparation of honey or lyophilized solutions (e.g., monodrug or extract) is that the dilution of honey is expressed as a percentage (%) instead of mg/mL or µg/mL. In this case, it is possible to determine this percentage by making proportions of honey and water or culture medium, as follows: weight/volume (*w*/*v*) or volume/volume (*v*/*v*). Although some authors prefer to use the *v*/*v* ratio [53,54], most of them perform the dilution based on the *w*/*v* ratio [24,55,56,57]. We recommend the use of *w*/*v* ratio due to the viscosity of the sample, which may cause substantial material loss during pipetting and errors.

Honeys from different botanical and geographical origins exhibit varying antimicrobial potency. Thus, they can be classified into different categories, of which monofloral honey seems to be the most promising and interesting type as a natural remedy. Manuka honey, a monofloral honey derived from the Manuka tree (*Leptospermum scoparium*), stands out for its antimicrobial and antioxidant properties. Manuka honey has been used as a parameter to comparatively classify the antimicrobial potency of other honey [58]. In general, dark colored honeys exhibit more expressive antimicrobial activity. The most potent honeys, such as Manuka, dark buckwheat, Heather, or molasses, have MIC values ranging from 1% to 12.5% (*w*/*v*). Light colored honeys, such as clover honey (pasture honey) and acacia or rapeseed honey, were found to be less potent, with MIC values ranging between 25% and 50% (*w*/*v*) [25]. These well-known honeys can be used as parameters to define whether a sample has strong, moderate, or weak antimicrobial activity. Thus, in Table 2, we propose some parameters for the determination of the antimicrobial potency of honeys, according to Albaridi’s review [25].

**Figure 3:**
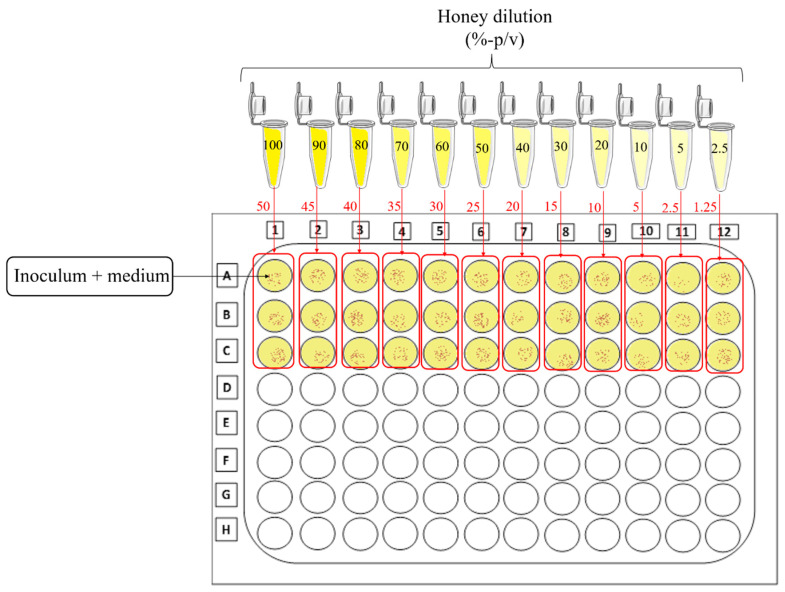
Schematic representation of the serial dilution of honey for antimicrobial testing. The honey sample should be diluted separately by preparing a solution with twice the concentration expected to be in the well of the microplate. After adding the diluted honey solution to the same amount of inoculum, the concentration will drop by half in the well. To obtain these concentrations, it is necessary to use the microplate horizontally, as demonstrated in the figure.

We suggest the preparation of an initial solution which should be serially diluted at a 1:2 ratio, since it would be the maximum concentration (50% *w*/*v*) to determine a discrete antimicrobial activity that is not due to lack of culture medium [25]. We do not recommend 1:2 dilutions in the wells of the microplate since the concentration drops very sharply from one well to the other. Instead, we suggest preparing individual solutions to be added together with the inoculum. This way, the concentration drops by half from the initial solutions and will have a difference of 5% from one well to the other in the 96-well plate, ranging from 50% to 1.25% (Figure 3).

In addition to determining the antimicrobial activity of honey against planktonic bacteria, it is essential to assess its potential against biofilms. Biofilms are dense communities that grow on inert surfaces and are embedded in self-secreting, high-molecular-weight polymers. When organisms form a biofilm, they can adapt to environmental changes by altering their gene expression and becoming highly resistant to antimicrobials [13].

As mentioned in Section 2 of this review, there is an association between the presence of oral biofilms and the onset and progression of oral diseases, such as caries and periodontal disease [59,60]. Thus, a drug or product with antimicrobial activity should necessarily have a positive antibiofilm activity against mature biofilms to be considered effective for the prevention and/or treatment of oral diseases.

An ideal and standardized in vitro assay to assess the effectiveness of antibiofilm agents has not been established yet, although different testing methods are available. Approaches such as the modified Robbins device, Calgary biofilm device, disc reactor, Centers for Disease Control (CDC) biofilm reactor, perfused biofilm fermenter, and model bladder have been considered promising and representative of in vivo conditions [61]. In addition, multispecies biofilm models better mimic what occurs in the oral cavity than single-species biofilm models. There is a wide range of tools available for biofilm analysis, from colony counting to more modern techniques, such as fluorescent biofilm labeling in conjunction with mathematical predictive modeling, such as COMSTAT [62]. For a quick and low-cost analysis, we commonly use the Calgary device to determine the antibiofilm activity of propolis samples [21] and suggest that it should be performed to assess the antibiofilm activity of honey. The Calgary device allows biofilms to grow on rods suspended from a lid that fits into a 96-well plate. Once the device is suspended in the culture medium with inoculum, it allows for actual biofilm formation without deposition of gravity [63].

Thus, for testing the antibiofilm activity of honey, we suggest diluting the sample as shown in Figure 3 and treating multispecies biofilms at concentrations greater than the MIC. Treatment intervals should be selected to simulate the daily consumption of honey by a person who substitutes the common sugar of their diet with this functional food.

## 5. Antimicrobial Activity of Honey against Oral Pathogens

Some literature reports show that honey is active against a wide range of pathogens associated with systemic infections, such as *Pseudomonas aeruginosa* [64], *Staphylococcus aureus* (MRSA) [65], *Escherichia coli, Proteus mirabilis, Staphylococcus aureus, Shigella flexneri,* and *Staphylococcus epidermidis* [66]. Testing the effectiveness of honey against planktonic bacteria is relevant, but the use of biofilm cultures in antimicrobial assays is highly recommended. Biofilm communities are embedded in self-producing polysaccharides, which creates a barrier that provides resistance to antimicrobials [18]. Some authors showed that multifloral honey has promising antibiofilm activity against systemic pathogens, such as *Pseudomonas aeruginosa*, *Staphylococcus aureus*, Methicillin-resistant *Staphylococcus aureus*, and *Escherichia coli* [39,67,68,69]. Honey samples should be ideally examined for their antimicrobial mechanism(s) of action, e.g., downregulation of virulence genes, disruption of the bacterial cell wall, interference in the formation or size of cells and/or accessory components (fimbriae and flagella), and damage to nucleic acids. The mechanisms of action of honey against systemic bacteria are well documented [5], but there are still gaps concerning the analysis of antibiofilm properties against oral bacteria. Thus, this comprehensive review brings an overview of all the evidence on the in vitro antimicrobial activity of honey against oral microorganisms. Bibliographical searches were carried out in the databases Medline via PubMed, SciVerse Scopus, Web of Science, SciELO, LILACS, Cochrane Library, and Google Scholar using the following descriptors: honey, antimicrobial activity, and oral bacterium (Table 3).

Although some papers show the honey’s botanical origin, not all manuscripts provide this information (Table 3). This may result in a gap in the literature that needs to be filled. To avoid that, future research on honey should pay attention to its botanical origin.

Most of the studies shown in Table 3 used the broth microdilution method for determining the antimicrobial activity of honey samples [70,72,73,74,75,76,77,78], and only a few studies used the agar diffusion method [55,71]. These findings are consistent with what was discussed in Section 4 of this review. Of all the studies presented in Table 3, only one examined aspects such as the mechanism of action, chemical composition, the relationship between chemical profile and bioactivity, as well as the antibiofilm activity of honey in mature biofilms [79]. On the other hand, none of the studies showed data indicating that honey can cause cell damage in bacteria or that it has antibiofilm activity against multispecies biofilms.

Although the antimicrobial activity of honey against microorganisms present in wounds is well documented [5], its effectiveness against oral microorganisms remains to be determined. Further research should establish the antimicrobial activity of honey against mature multispecies oral biofilms [18].

In addition to the in vitro studies summarized in Table 3, the literature presents some clinical trials that have evaluated the antimicrobial effectiveness of honey against oral diseases. Because it is a food and not a drug, clinical trials with honey can be ethically less complex. However, this does not diminish the importance of carrying out in vitro studies for the classification, characterization, and definition of the antimicrobial mechanisms of action. Despite the advances, the selected clinical trials have important limitations to consider, as further discussed herein.

A comparative, in vitro, and clinical study was performed to determine the antimicrobial activity of 0.2% chlorhexidine and a honey-containing mouthwash. The in vitro phase of the study was carried out using the agar diffusion method, whereas the second phase consisted of a randomized blind clinical trial, with a total sample size of 66 individuals. The in vitro data showed that the honey mouthwash effectively inhibited the growth of *Eubacterium nodatum*, *Campylobacter rectus*, *Streptococcus mutans*, *Aggregatibacter actinomycetemcomitans*, *Porphyromonas gingivalis*, and *Streptococcus sanguinis*, although 0.2% chlorhexidine was more effective. In the clinical trial, the intergroup analysis between chlorhexidine and honey showed that both formulations significantly reduced plaque formation (*p* < 0.001). Although chlorhexidine was more effective than the honey-containing mouthwash, there was no significant difference between them (*p* = 0.670) [80]. This study showed the in vitro and clinical effectiveness of a honey formulation in reducing the growth of oral microorganisms, supporting the hypothesis that honey can be used as a protective food against biofilm-dependent oral diseases. However, the in vitro data lack important pharmacological information, such as the definition of the mechanisms of action and the chemical profile of the sample. Furthermore, the clinical trial lacks important indices for periodontal disease assessment, such as clinical parameters and/or salivary cytokine quantification.

Another study investigated the effectiveness of Manuka honey in reducing clinical levels of biofilm and gingivitis. The authors developed a “honey strip” chewable formulation. The study was randomized and had a sample size of 30 participants, who were allocated into two groups: one group chewed the Manuka product and the other chewed a sugar-free gum. The chewing time was 10 min, three times a day, after each meal. Plaque and gingivitis scores were recorded before and after 21 days. In the Manuka honey group, there were statistically significant reductions in mean plaque scores (from 10.99 to 0.65, *p* = 0.001) and in the number of bleeding sites (from 48% to 17%, *p* = 0.001). In contrast, no significant differences were observed in the control group [81]. This showed that Manuka honey is as effective against oral microorganisms as it is against systemic bacteria, as demonstrated in previous clinical studies. The authors also examined the pattern of gingival bleeding, which is an important diagnostic index for periodontal diseases. While these findings correspond to a pilot study, they provide important evidence for the use of honey to prevent biofilm-dependent oral diseases or even as an alternative therapy. However, aspects such as the mechanism of action, dosage, and therapeutic indications need to be studied before honey formulations can be indicated as a therapy in dentistry.

The antibacterial activity of commercial honey and green tea solutions was previously assessed based on salivary *Streptococcus mutans* counts. Thirty healthy individuals were randomly allocated into two groups of 15, one for each solution. Saliva samples were collected before and after rinsing the test solutions in both groups. The results showed that after a single mouth rinsing, the count of colony-forming units (CFU/mL) decreased from 2.28 × 10^8^ to 5.64 × 10^7^ CFU/mL in the honey group and from 1.95 × 10^9^ to 2.9 × 10^8^ in the green tea group. There was a statistically significant difference for both groups in relation to baseline CFU counts [82].

A similar study assessed the effects of Manuka honey on the count of salivary streptococci [83]. Although the study showed positive results, the authors did not measure the antibiofilm activity of Manuka honey nor included any clinical parameters in the analysis. However, collectively, the study shows that honey significantly modulates the microaerophilic microbiota of the mouth.

Recently, a clinical trial evaluated toothpaste’s efficacy composed of natural ingredients, including honey from Apis mellifera bees. After six months of using the tested toothpaste, patients enrolled in this study presented reductions of 23.5%, 25.6%, and 73.3% for dental plaque, gingival index, and bleeding index outcomes, respectively [84]. Although not determined for oral bacteria, the antimicrobial mechanisms of honey include inhibition of membrane and intracellular proteins with subsequent DNA damage [85].

Taken together, the literature, including recent manuscripts, classifies honey as a functional food with antimicrobial activity [84,85,86,87,88]. However, honey’s clinical efficacy and antibacterial mechanisms against oral biofilms remain poorly understood and underexplored; just a few manuscripts addresses it [84]. Thus, further in vitro and in vivo studies are needed to support the use of honey as an antimicrobial and/or as adjuvant therapy in dental practice.

## 6. Concluding Remarks

Mounting evidence has shown that honey has promising antimicrobial activity. This functional food has been studied and used in folk medicine for many centuries to treat infections, particularly those associated with wounds and bedsores. Nevertheless, only a few scientific studies have investigated the use of honey to prevent and/or treat biofilm-dependent oral diseases such as periodontitis. The antimicrobial activities of honey against oral microorganisms, as well as its antibiofilm activity against mature biofilms, mechanism of action, chemical composition, and clinical indications, remain largely unknown.

Honey has considerable potential to be used as an alternative therapy for biofilm-dependent oral diseases and/or as an auxiliary functional food for maintaining health. However, further research is needed to demonstrate this activity in mature oral biofilms, as well as the associated chemical pathways and the molecular targets in the bacterial cell. Filling these gaps may provide evidence to recommend the rational consumption of honey instead of common sugar for oral and systemic health benefits. Moreover, the promising antibiofilm properties of honey may encourage the development of clinical trials aimed at using honey products as a complementary therapy in dental care.

## Figures and Tables

**Table 1:** Factors related to the antimicrobial activity of honey.

Component/Physical Characteristic	Considerations about the Antimicrobial Activity of Honey	References
Low pH	Due to the high concentration of organic acids (mainly gluconic acid), most honey samples have a pH ranging between 3.4 and 6.0 which, in combination with high osmotic pressure, can eliminate and/or prevent microbial colonization.	[25]
Osmotic effect	High osmolarity has been shown to play a role in the antimicrobial properties of honey. Despite this, high concentrations of glucose alone showed no inhibitory effect on bacterial growth. Thus, sugar-induced osmolarity in honey is only considered an adjuvant that provides an unfavorable environment for pathogens.	[26]
Hydrogen peroxide (H_2_O_2_)	Hydrogen peroxide (H_2_O_2_) levels are directly proportional to the antibacterial activity of honey. Therefore, H_2_O_2_ is considered a predictive biomarker of its antibacterial activity. Glucose oxidase is an enzyme present in honey that is activated when the honey is diluted. This enzyme acts on endogenous glucose to produce hydrogen peroxide. H_2_O_2_ is involved with oxidative damage, causing inhibition of bacterial growth and DNA degradation. However, these effects are synergistically modulated by other components present in honey, since higher concentrations of pure H_2_O_2_ are needed to obtain similar effects as compared to the low H_2_O_2_ levels present in honey samples.	[25,27,28]
Phenolic compounds	The therapeutic effect of honey is attributed to the presence of several antioxidants, including phenolic compounds, such as flavonoids and phenolic acids. Some phenolic compounds, such as pinocembrin and serum acid, have been strongly associated with the antimicrobial activity of honey. Nevertheless, information on the mechanism of action and effective doses of these compounds is yet to be determined.	[2,29]
Methylglyoxal (MGO)	MGO is an organic compound derived from dihydroxyacetone. The presence of MGO in honey contributes to its antimicrobial activity, even in honeys with low levels of peroxide (e.g., Manuka honey). MGO causes loss of membrane integrity and changes the structure of bacterial fimbriae and flagella, which impairs microbial adhesion and motility.	[30]
Bee peptides (Defensin-1)	Defensin-1 is a peptide secreted by the hypopharyngeal glands of bees. This peptide is active against Gram-positive bacteria, including *Bacillus subtilis* and *Staphylococcus aureus*. Although bees produce other peptides, only defensin-1 has been detected in honey and was found to have antimicrobial activity.	[31]

**Table 2:** Parameters for determination of the antimicrobial potency of honey samples.

MIC (%, *w*/*v*)	Antimicrobial Activity
1.0% to 12.5%	Strong
12.5% to 50.0%	Moderate
>50.0%	Weak

Adapted from Albaridi, 2019 [25].

**Table 3:** In vitro studies of antimicrobial activity of honey against oral microorganisms.

Type of Honey (Plant Origin)	Strain	Method	Results	Reference
Honey from central Switzerland, honey from the German plain, and Manuka honey (*Leptospermum scoparium*).	*Streptococcus gordonii*, *Streptococcus sanguinis*, *Streptococcus mutans*, *Streptococcus sobrinus*, *Lactobacillus acidophilus*, *Actinomyces naeslundii.*	Antimicrobial activity (MIC determination) and anti-adherent activity by counting colony-forming units per mL (CFU/mL).	The three honey samples inhibited the specific growth of oral bacterial strains. When using a multispecies biofilm model, none of the samples were significantly effective.	[70]
Kerala commercial honey, India.	*Streptococcus mutans*	Antimicrobial activity determined by the agar diffusion method.	A discrete zone of inhibition of the growth of *Streptococcus mutans* was observed.	[71]
Manuka honey and white clover honey (*Trifolium repens)*.	*Staphylococcus aureus*, *Escherichia coli*, *Streptococcus mutans*, *Streptococcus sobrinus*, *Streptococcus sanguinis*, *Streptococcus gordonii*, *Fusobacterium nucleatum*, *Porphyromonas gingivalis*, and *Prevotella intermedia*	Antimicrobial activity by broth microdilution for determination of MIC and MBC values.	Both honeys inhibited most of the tested stains, except *Streptococcus mutans.* Manuka honey displayed slightly greater inhibitory efficacy, with MICs ranging between 6.3% and 25%, whereas the MICs of clover honey ranged from 6.3% to 50%. Honeys with neutral pH had little antimicrobial activity.	[72]
Swiss multifloral honey, Manuka honey NPA 5+, Manuka honey NPA 15+ (*Leptospermum scoparium)*, Manuka honey label “MGO 400+” (*Leptospermum scoparium)*, equivalent to NPA 20+, Manuka honey NPA 25+, MediHoneyTM medicinal honey, and MediHoneyTM gel sheet.	*Aggregatibacter* *actinomycetemcomitans*, *Porphyromonas gingivalis*, and *Streptococcus mutans*	Initial screening for antimicrobial activity by the agar diffusion method. The most active samples were selected for the determination of MIC and MBC values.	Manuka honey below an NPA value of 15 showed the least potential to inhibit bacterial growth, even less—although not significantly—than Swiss multifloral honey. Manuka honey above an NPA value of 15 showed a significantly greater antibacterial effect compared to the other honeys tested. All Manuka honey preparations were more effective in inhibiting the growth of *Porphyromonas gingivalis* and *Aggregatibacter* *actinomycetemcomitans* as compared to *S. mutans*.	[73]
Manuka honey 1 and 2 (*Leptospermum scoparium*).	*Streptococcus mutans*, *Streptococcus sobrinus*, *Lactobacillus rhamnosus*, *Actinomyces viscosus*, *Porphyromonas gingivalis*, and *Fusobacterium nucleatum.*	Antimicrobial activity (MIC determination) and anti-adherent activity by counting colony-forming units per mL (CFU/mL).	The antibacterial activity of Manuka 1 was the most important. The two honeys tested showed a weak ability to inhibit the adhesion of *S. mutans* cells onto a glass surface at sub-MIC concentrations. Manuka 1 completely inhibited multispecies biofilm formation at a concentration of 200 μg/mL. Manuka 2 inhibited biofilm formation weakly at a concentration of 200 μg/mL, but strongly at a concentration of 500 μg/mL.	[74]
Eucalyptus honey (*Eucalyptus cladocalyx*).	*Steptococcus mutans*, *Steptococcus sobrinus*, *Steptococcus gordonii*, *Steptococcus salivarius*, *Steptococcus sanguinis*, *Steptococcus anginosus*, *Steptococcus oralis*, and *Escherichia coli*.	Antimicrobial activity by broth microdilution for determination of MIC values.	Eucalyptus honey had MIC of 25% (*v*/*v*) on the tested strains, except for *Streptococcus anginosus* and *Streptococcus oralis*, whose MIC values were 17% (*v*/*v*) and 12% (*v*/*v*), respectively. Hypertonic sugar control had MIC of 25% (*vol*/*vol*) on all bacterial strains.	[75]
Manuka honey (*Leptospermum scoparium*), eucalyptus honey (*Eucalyptus cladocalyx*), pin cushion honey (*Leucospermum cordifolium*), and Erica honey (*Erica* species—Fynbos).	*Streptococcus mutans*, *Streptococcus salivarius*, *Streptococcus sanguis*, *Streptococcus anginosus*, *Streptococcus gordonii*, *Streptococcus oralis*, *Streptococcus sobrinus*, *Candida albicans*, *Escherichia coli* and *Staphylococcus aureus.*	Antimicrobial activity by broth microdilution for determination of MIC values.	*Candida albicans* yeast (MIC of 40%) was more resistant to the tested honeys than were the bacterial strains. *Streptococcus anginosus* (MIC of 17%) and *S. oralis* (MIC of 12.5%) were more sensitive to honey than the other strains. The honey samples showed MIC of 25% against other oral streptococci.	[76]
Azarian honey.	*Staphylococcus aureus*, *Staphylococcus epidermidis*, *Streptococcus mutans*, MRSA, and *Enterococcus faecalis*	Antimicrobial activity by broth microdilution for the determination of MIC values.	The lowest MIC value of the tested honeys was found for *Staphylococcus aureus*. The MIC of honey for *Escherichia coli* was higher than that for the other strains. The mean MIC for *Staphylococcus epidermidis* was similar to that of *Staphylococcus aureus*. The MBC values for *S. mutans*, MRSA, *Staphylococcus aureus*, *Staphylococcus epidermidis*, and *Enterococcus faecalis* were 7.81%, 8.52%, 7.55%, 12.03%, and 7.81% (*v*/*v*), respectively. The combination of propolis and honey reduced the MIC for all bacterial strains.	[77]
Commercial honey from Saudi Arabia (Langnese Honig, Germany).	*Streptococcus mutans*	Antimicrobial activity (microdilution method) and inhibition of biofilm formation.	Natural honey reduced *Streptococcus mutans* growth more effectively than artificial honey (control) at the concentrations of 25% and 12.5%. At 50% and 25%, both honeys significantly reduced bacterial growth and biofilm formation as compared to the TSB control. Natural honey was also able to decrease the maximum growth rate of *Streptococcus mutans* compared to artificial honey.	[78]
Ramadan natural honey.	*Streptococcus mutans*	Antimicrobial activity by the agar diffusion method.	Significant antibacterial activity was detected against *Streptococcus mutans* at concentrations greater than 20% and against *Lactobacillus* at a concentration of 100%.	[55]
Manuka honey (*Leptospermum scoparium*) and commercial multifloral honey from Germany.	*Porphyromonas gingivalis*	Antimicrobial activity by the microdilution method (determination of the MIC) and antibiofilm activity.	Manuka honey and commercial honey inhibited 50% of *Porphyromonas gingivalis* growth at concentrations of 2% and 5%, respectively. Manuka honey contained 1.87 mg/kg of hydrogen peroxide, whereas the commercial honey had 3.74 mg/kg. The amount of methylglyoxal was 2 mg/kg in the domestic honey and 982 mg/kg in Manuka honey. At 10%, both types of honey inhibited *Porphyromonas gingivalis* biofilm formation and reduced the number of viable bacteria in 42-hour-old biofilms.	[79]

## References

[1] Da Silva P.M., Gauche C., Gonzaga L.V., Costa A.C.O., Fett R. (2016). Honey: Chemical Composition, Stability and Authenticity. Food Chem..

[2] Cianciosi D., Forbes-Hernández T., Afrin S., Gasparrini M., Reboredo-Rodriguez P., Manna P., Zhang J., Bravo Lamas L., Martínez Flórez S., Agudo Toyos P. (2018). Phenolic Compounds in Honey and Their Associated Health Benefits: A Review. Molecules.

[3] Alqarni A.S., Owayss A.A., Mahmoud A.A. (2016). Physicochemical Characteristics, Total Phenols and Pigments of National and International Honeys in Saudi Arabia. Arab. J. Chem..

[4] Konstantinidi M., Koutelidakis A.E. (2019). Functional Foods and Bioactive Compounds: A Review of Its Possible Role on Weight Management and Obesity’s Metabolic Consequences. Medicines.

[5] Cooper R. (2016). Honey for Wound Care in the 21st Century. J. Wound Care.

[6] Abd El-Malek F.F., Yousef A.S., El-Assar S.A. (2017). Hydrogel Film Loaded with New Formula from Manuka Honey for Treatment of Chronic Wound Infections. J. Glob. Antimicrob. Resist..

[7] Molan P., Rhodes T. (2015). Honey: A Biologic Wound Dressing. Wounds.

[8] Dreyfus J., Delhougne G., James R., Gayle J., Waycaster C. (2018). Clostridial Collagenase Ointment and Medicinal Honey Utilization for Pressure Ulcers in US Hospitals. J. Med. Econ..

[9] Colombo A.P.V., do Souto R.M., da Silva-Boghossian C.M., Miranda R., Lourenço T.G.B. (2015). Microbiology of Oral Biofilm-Dependent Diseases: Have We Made Significant Progress to Understand and Treat These Diseases?. Curr. Oral Health Rep..

[10] Roberts A.P., Mullany P. (2010). Oral Biofilms: A Reservoir of Transferable, Bacterial, Antimicrobial Resistance. Expert Rev. Anti-Infect. Ther..

[11] Vieira Colombo A.P., Magalhães C.B., Hartenbach F.A.R.R., Martins do Souto R., Maciel da Silva-Boghossian C. (2016). Periodontal-Disease-Associated Biofilm: A Reservoir for Pathogens of Medical Importance. Microb. Pathog..

[12] Ozen A.E., Pons A., Tur J.A. (2012). Worldwide Consumption of Functional Foods: A Systematic Review. Nutr. Rev..

[13] Berger D., Rakhamimova A., Pollack A., Loewy Z. (2018). Oral Biofilms: Development, Control, and Analysis. High-Throughput.

[14] Štšepetova J., Truu J., Runnel R., Nõmmela R., Saag M., Olak J., Nõlvak H., Preem J.K., Oopkaup K., Krjutškov K. (2019). Impact of Polyols on Oral Microbiome of Estonian Schoolchildren. BMC Oral Health.

[15] Jepsen S., Blanco J., Buchalla W., Carvalho J.C., Dietrich T., Dörfer C., Eaton K.A., Figuero E., Frencken J.E., Graziani F. (2017). Prevention and Control of Dental Caries and Periodontal Diseases at Individual and Population Level: Consensus Report of Group 3 of Joint EFP/ORCA Workshop on the Boundaries between Caries and Periodontal Diseases. J. Clin. Periodontol..

[16] Lamont R.J., Koo H., Hajishengallis G. (2018). The Oral Microbiota: Dynamic Communities and Host Interactions. Nat. Rev. Microbiol..

[17] Marsh P.D. (2006). Dental Plaque as a Biofilm and a Microbial Community—Implications for Health and Disease. BMC Oral Health.

[18] Marsh P.D. (2004). Dental Plaque as a Microbial Biofilm. Caries Res..

[19] Socransky S.S., Haffajee A.D., Cugini M.A., Smith C., Kent R.L. (1998). Microbial Complexes in Subgingival Plaque. J. Clin. Periodontol..

[20] Haffajee A.D., Socransky S.S., Patel M.R., Song X. (2008). Microbial Complexes in Supragingival Plaque. Oral Microbiol. Immunol..

[21] Miranda S.L.F., Damasceno J.T., Faveri M., Figueiredo L., da Silva H.D., de Alencar S.M.A., Rosalen P.L., Feres M., Bueno-Silva B. (2019). Brazilian Red Propolis Reduces Orange-Complex Periodontopathogens Growing in Multispecies Biofilms. Biofouling.

[22] Lu L., Hu W., Tian Z., Yuan D., Yi G., Zhou Y., Cheng Q., Zhu J., Li M. (2019). Developing Natural Products as Potential Anti-Biofilm Agents. Chin. Med..

[23] Israili Z.H. (2014). Antimicrobial Properties of Honey. Am. J. Ther..

[24] Allen K.L., Molan P.C., Reid G.M. (1991). A Survey of the Antibacterial Activity of Some New Zealand Honeys. J. Pharm. Pharmacol..

[25] Albaridi N.A. (2019). Antibacterial Potency of Honey. Int. J. Microbiol..

[26] Matzen R.D., Zinck Leth-Espensen J., Jansson T., Nielsen D.S., Lund M.N., Matzen S. (2018). The Antibacterial Effect In Vitro of Honey Derived from Various Danish Flora. Dermatol. Res. Pract..

[27] Brudzynski K., Lannigan R. (2012). Mechanism of Honey Bacteriostatic Action against MRSA and VRE Involves Hydroxyl Radicals Generated from Honey’s Hydrogen Peroxide. Front. Microbiol..

[28] Bucekova M., Buriova M., Pekarik L., Majtan V., Majtan J. (2018). Phytochemicals-Mediated Production of Hydrogen Peroxide Is Crucial for High Antibacterial Activity of Honeydew Honey. Sci. Rep..

[29] Agbaje E.O., Ogunsanya T., Aiwerioba O.I.R. (2006). Conventional use of honey as antibacterial agent. Ann. Afr. Med..

[30] Rabie E., Serem J.C., Oberholzer H.M., Gaspar A.R.M., Bester M.J. (2016). How Methylglyoxal Kills Bacteria: An Ultrastructural Study. Ultrastruct. Pathol..

[31] Szweda P., Toledo V.A.A. (2016). Antimicrobial Activity of Honey. Honey Analysis.

[32] Escuredo O., Dobre I., Fernández-González M., Seijo M.C. (2014). Contribution of Botanical Origin and Sugar Composition of Honeys on the Crystallization Phenomenon. Food Chem..

[33] Molan P.C., Allen K.L. (1996). The Effect of Gamma-Irradiation on the Antibacterial Activity of Honey. J. Pharm. Pharmacol..

[34] Mandal M.D., Mandal S. (2011). Honey: Its Medicinal Property and Antibacterial Activity. Asian Pac. J. Trop. Biomed..

[35] Adams C.J., Boult C.H., Deadman B.J., Farr J.M., Grainger M.N.C., Manley-Harris M., Snow M.J. (2008). Isolation by HPLC and Characterisation of the Bioactive Fraction of New Zealand Manuka (Leptospermum Scoparium) Honey. Carbohydr. Res..

[36] Mavric E., Wittmann S., Barth G., Henle T. (2008). Identification and Quantification of Methylglyoxal as the Dominant Antibacterial Constituent of Manuka (Leptospermum Scoparium) Honeys from New Zealand. Mol. Nutr. Food Res..

[37] Carter D.A., Blair S.E., Cokcetin N.N., Bouzo D., Brooks P., Schothauer R., Harry E.J. (2016). Therapeutic Manuka Honey: No Longer so Alternative. Front. Microbiol..

[38] Packer J.M., Irish J., Herbert B.R., Hill C., Padula M., Blair S.E., Carter D.A., Harry E.J. (2012). Specific Non-Peroxide Antibacterial Effect of Manuka Honey on the *Staphylococcus aureus* Proteome. Int. J. Antimicrob. Agents.

[39] Bucekova M., Jardekova L., Juricova V., Bugarova V., Di Marco G., Gismondi A., Leonardi D., Farkasovska J., Godocikova J., Laho M. (2019). Antibacterial Activity of Different Blossom Honeys: New Findings. Molecules.

[40] Sakihama Y., Cohen M.F., Grace S.C., Yamasaki H. (2002). Plant Phenolic Antioxidant and Prooxidant Activities: Phenolics-Induced Oxidative Damage Mediated by Metals in Plants. Toxicology.

[41] Brudzynski K., Abubaker K., St-Martin L., Castle A. (2011). Re-Examining the Role of Hydrogen Peroxide in Bacteriostatic and Bactericidal Activities of Honey. Front. Microbiol..

[42] Brudzynski K. (2006). Effect of Hydrogen Peroxide on Antibacterial Activities of Canadian Honeys. Can. J. Microbiol..

[43] Kuś P.M., Szweda P., Jerković I., Tuberoso C.I.G. (2016). Activity of Polish Unifloral Honeys against Pathogenic Bacteria and Its Correlation with Colour, Phenolic Content, Antioxidant Capacity and Other Parameters. Lett. Appl. Microbiol..

[44] Kwakman P.H.S., Zaat S.A.J. (2012). Antibacterial Components of Honey. IUBMB Life.

[45] Ramos F.A., Takaishi Y., Shirotori M., Kawaguchi Y., Tsuchiya K., Shibata H., Higuti T., Tadokoro T., Takeuchi M. (2006). Antibacterial and Antioxidant Activities of Quercetin Oxidation Products from Yellow Onion (Allium Cepa) Skin. J. Agric. Food Chem..

[46] Brudzynski K., Miotto D., Kim L., Sjaarda C., Maldonado-Alvarez L., Fukś H. (2017). Active Macromolecules of Honey Form Colloidal Particles Essential for Honey Antibacterial Activity and Hydrogen Peroxide Production. Sci. Rep..

[47] Lu J., Carter D.A., Turnbull L., Rosendale D., Hedderley D., Stephens J., Gannabathula S., Steinhorn G., Schlothauer R.C., Whitchurch C.B. (2013). The Effect of New Zealand Kanuka, Manuka and Clover Honeys on Bacterial Growth Dynamics and Cellular Morphology Varies According to the Species. PLoS ONE.

[48] Henriques A.F., Jenkins R.E., Burton N.F., Cooper R.A. (2010). The Intracellular Effects of Manuka Honey on Staphylococcus aureus. Eur. J. Clin. Microbiol. Infect. Dis..

[49] Almasaudi S. (2021). The Antibacterial Activities of Honey. Saudi J. Biol. Sci..

[50] Anthimidou E., Mossialos D. (2013). Antibacterial Activity of Greek and Cypriot Honeys Against *Staphylococcus aureus* and *Pseudomonas Aaeruginosa* in Comparison to Manuka Honey. J. Med. Food.

[51] Balouiri M., Sadiki M., Ibnsouda S.K. (2016). Methods for in vitro Evaluating Antimicrobial Activity: A Review. J. Pharm. Anal..

[52] Pfaller M.A., Sheehan D.J., Rex J.H. (2004). Determination of Fungicidal Activities against Yeasts and Molds: Lessons Learned from Bactericidal Testing and the Need for Standardization. Clin. Microbiol. Rev..

[53] Almasaudi S.B., Al-Nahari A.A.M., Abd El-Ghany E.S.M., Barbour E., Al Muhayawi S.M., Al-Jaouni S., Azhar E., Qari M., Qari Y.A., Harakeh S. (2017). Antimicrobial Effect of Different Types of Honey on *Staphylococcus aureus*. Saudi J. Biol. Sci..

[54] Mama M., Teshome T., Detamo J. (2019). Antibacterial Activity of Honey against Methicillin-Resistant *Staphylococcus aureus*: A Laboratory-Based Experimental Study. Int. J. Microbiol..

[55] Ahmadi-Motamayel F., Hendi S.S., Alikhani M.Y., Khamverdi Z. (2013). Antibacterial Activity of Honey on Cariogenic Bacteria. J. Dent. Tehran.

[56] Johnston M., McBride M., Dahiya D., Owusu-Apenten R., Singh Nigam P. (2018). Antibacterial Activity of Manuka Honey and Its Components: An Overview. AIMS Microbiol..

[57] Anand S., Deighton M., Livanos G., Morrison P.D., Pang E.C.K., Mantri N. (2019). Antimicrobial Activity of Agastache Honey and Characterization of Its Bioactive Compounds in Comparison with Important Commercial Honeys. Front. Microbiol..

[58] Alvarez-Suarez J., Gasparrini M., Forbes-Hernández T., Mazzoni L., Giampieri F. (2014). The Composition and Biological Activity of Honey: A Focus on Manuka Honey. Foods.

[59] Sanz M., Beighton D., Curtis M.A., Cury J.A., Dige I., Dommisch H., Ellwood R., Giacaman R.A., Herrera D., Herzberg M.C. (2017). Role of Microbial Biofilms in the Maintenance of Oral Health and in the Development of Dental Caries and Periodontal Diseases. Consensus Report of Group 1 of the Joint EFP/ORCA Workshop on the Boundaries between Caries and Periodontal Disease. J. Clin. Periodontol..

[60] Colombo A.P.V., Tanner A.C.R. (2019). The Role of Bacterial Biofilms in Dental Caries and Periodontal and Peri-Implant Diseases: A Historical Perspective. J. Dent. Res..

[61] Kırmusaoğlu S., Kırmusaoğlu S. (2019). The Methods for Detection of Biofilm and Screening Antibiofilm Activity of Agents. Antimicrobials, Antibiotic Resistance, Antibiofilm Strategies and Activity Methods.

[62] Wilson C., Lukowicz R., Merchant S., Valquier-Flynn H., Caballero J., Sandoval J., Okuom M., Huber C., Brooks T.D., Wilson E. (2017). Quantitative and Qualitative Assessment Methods for Biofilm Growth: A Mini-Review. Res. Rev. J. Eng. Technol..

[63] Ceri H., Olson M.E., Stremick C., Read R.R., Morck D., Buret A. (1999). The Calgary Biofilm Device: New Technology for Rapid Determination of Antibiotic Susceptibilities of Bacterial Biofilms. J. Clin. Microbiol..

[64] Yang C., Mavelli G.V., Nacharaju P., Li K., Cleare L.G., Nosanchuk J.D., Friedman J.M., Abuzeid W.M. (2020). Novel Nitric Oxide–generating Platform Using Manuka Honey as an Anti-biofilm Strategy in Chronic Rhinosinusitis. Int. Forum Allergy Rhinol..

[65] Cremers N., Belas A., Santos Costa S., Couto I., de Rooster H., Pomba C. (2020). In Vitro Antimicrobial Efficacy of Two Medical Grade Honey Formulations against Common High-risk Meticillin-resistant Staphylococci and *Pseudomonas* Spp. Pathogens. Vet. Dermatol..

[66] Ghramh H.A., Khan K.A., Alshehri A.M.A. (2019). Antibacterial Potential of Some Saudi Honeys from Asir Region against Selected Pathogenic Bacteria. Saudi J. Biol. Sci..

[67] Oliveira A., Ribeiro H.G., Silva A.C., Silva M.D., Sousa J.C., Rodrigues C.F., Melo L.D.R., Henriques A.F., Sillankorva S. (2017). Synergistic Antimicrobial Interaction between Honey and Phage against *Escherichia coli* Biofilms. Front. Microbiol..

[68] Hannan A., Bajwa A.E., Riaz S., Arshad U., Saleem S., Bajwa U.I. (2018). *In vitro Salmonella typhi* Biofilm Formation on Gallstones and Its Disruption by Manuka Honey. Pak. J. Pharm. Sci..

[69] Morroni G., Alvarez-Suarez J.M., Brenciani A., Simoni S., Fioriti S., Pugnaloni A., Giampieri F., Mazzoni L., Gasparrini M., Marini E. (2018). Comparison of the Antimicrobial Activities of Four Honeys from Three Countries (New Zealand, Cuba, and Kenya). Front. Microbiol..

[70] Habluetzel A., Schmid C., Carvalho T.S., Lussi A., Eick S. (2018). Impact of Honey on Dental Erosion and Adhesion of Early Bacterial Colonizers. Sci. Rep..

[71] Mathai K., Anand S., Aravind A., Dinatius P., Krishnan A.V., Mathai M. (2017). Antimicrobial Effect of Ginger, Garlic, Honey, and Lemon Extracts on *Streptococcus mutans*. J. Contemp. Dent. Pract..

[72] Safii S.H., Tompkins G.R., Duncan W.J. (2017). Periodontal Application of Manuka Honey: Antimicrobial and Demineralising Effects In Vitro. Int. J. Dent..

[73] Schmidlin P.R., English H., Duncan W., Belibasakis G.N., Thurnheer T. (2014). Antibacterial Potential of Manuka Honey against Three Oral Bacteria in Vitro. Swiss Dent. J..

[74] Badet C., Quero F. (2011). The *in vitro* Effect of Manuka Honeys on Growth and Adherence of Oral Bacteria. Anaerobe.

[75] Basson N.J., du Toit I.J., Grobler S.R. (1994). Antibacterial Action of Honey on Oral Streptococci. J. Dent. Assoc. S. Afr..

[76] Basson N.J., Grobler S.R. (2008). Antimicrobial Activity of Two South African Honeys Produced from Indigenous Leucospermum Cordifolium and Erica Species on Selected Micro-Organisms. BMC Complement. Altern. Med..

[77] Eslami H., Ariamanesh N., Ariamanesh A., Kafil H.S. (2016). Synergistic Effect of Honey and Azarian Propolis on Oral Microorganisms: An *in vitro* Study. J. Adv. Oral Res..

[78] Nassar H.M., Li M., Gregory R.L. (2012). Effect of Honey on *Streptococcus mutans* Growth and Biofilm Formation. Appl. Environ. Microbiol..

[79] Eick S., Schäfer G., Kwieciński J., Atrott J., Henle T., Pfister W. (2014). Honey—A Potential Agent against Porphyromonas Gingivalis: An *in vitro* Study. BMC Oral Health.

[80] Aparna S., Srirangarajan S., Malgi V., Setlur K.P., Shashidhar R., Setty S., Thakur S. (2012). A Comparative Evaluation of the Antibacterial Efficacy of Honey *in vitro* and Antiplaque Efficacy in a 4-Day Plaque Regrowth Model in vivo: Preliminary Results. J. Periodontol..

[81] English H.K.P., Pack A.R.C., Molan P.C. (2004). The Effects of Manuka Honey on Plaque and Gingivitis: A Pilot Study. J. Int. Acad. Periodontol..

[82] Abdelmegid F., Al-Agamy M., Alwohaibi A., Ka’abi H., Salama F. (2015). Effect of Honey and Green Tea Solutions on Streptococcus mutans. J. Clin. Pediatr. Dent..

[83] Rupesh S., Winnier J., Nayak U., Rao A., Reddy N., Peter J. (2014). Evaluation of the Effects of Manuka Honey on Salivary Levels of mutans Streptococci in Children: A Pilot Study. J. Indian Soc. Pedod. Prev. Dent..

[84] Nandlal B., Sreenivasan P.K., Shashikumar P., Devishree G., Bettahalli Shivamallu A. (2021). A randomized clinical study to examine the oral hygiene efficacy of a novel herbal toothpaste with zinc over a 6-month period. Int. J. Dent. Hyg..

[85] Al-Sayaghi A.M., Al-Kabsi A.M., Abduh M.S., Saghir S.A.M., Alshawsh M.A. (2022). Antibacterial Mechanism of Action of Two Types of Honey against Escherichia coli through Interfering with Bacterial Membrane Permeability, Inhibiting Proteins, and Inducing Bacterial DNA Damage. Antibiotics.

[86] Otreba M., Marek L., Tyczynska N., Stojko J., Rzepecka-Stojko A. (2021). Bee Venom, Honey, and Royal Jelly in the Treatment of Bacterial Infections of the Oral Cavity: A Review. Life.

[87] Deglovic J., Majtanova N., Majtan J. (2022). Antibacterial and Antibiofilm Effect of Honey in the Prevention of Dental Caries: A Recent Perspective. Foods.

[88] Prince A., Roy S., McDonald D. (2022). Exploration of the Antimicrobial Synergy between Selected Natural Substances on Streptococcus mutans to Identify Candidates for the Control of Dental Caries. Microbiol. Spectr..

